# LeishMicrosatDB: open source database of repeat sequences detected in six fully sequenced *Leishmania* genomes

**DOI:** 10.1093/database/bau078

**Published:** 2014-08-14

**Authors:** Manas R. Dikhit, Kanhu C. Moharana, Bikash R. Sahoo, Ganesh C. Sahoo, Pradeep Das

**Affiliations:** ^1^Biomedical Informatics Center and ^2^Department of Molecular Biology, Rajendra Memorial Research Institute of Medical Sciences, Patna 800007, India

## Abstract

A *Leishmania* Microsatellite Database (LeishMicrosatDB) is reported for genome wise mining of microsatellites in six *Leishmania* species, using *in silico* techniques. This was created to provide parasitologists a platform to understand the genome characterization, mapping, phylogeny and evolutionary analysis. The present version of the database contains 1 738 669 simple sequence repeats of which 181 s756 repeats are present in compound form. The repeats can be sought in a chromosome using input parameters such as repeat type (mono- hexa), coding status, repeat unit length and repeat sequence motif. The genic repeats have been further hyperlinked with their corresponding locus id, and the database is appended with primer3 plus for primer designing of selected repeats with left and right flanking sequences up to 250 bp. Information on clustering and polymorphic repeats can also be retrieved. This database may also be adopted as a tool to study the relative occurrence and distribution of microsatellites across the parasitic genome. The database can enable a biologist to select markers at desired intervals over the chromosomes, and can be accessed as an open source repository at http://biomedinformri.com/leishmicrosat.

**Database URL:** http://biomedinformri.com/leishmicrosat

## Introduction

*Leishmania* is a genus of protozoan parasites that infect macrophages causing a broad spectrum of diseases, ranging from self-limiting cutaneous leishmaniasis to severe mucocutaneous leishmaniasis with fatal spontaneous evolution. Leishmaniasis comprises a group of diseases having extensive morbidity and mortality in most developing countries. Infection with pathogenic *Leishmania* results an annual incidence of 2 million cases in 88 countries (www.who.int/tdr/disease/leish). Molecular markers are highly necessary to identify different strain through human populations, and identify animal reservoirs of the strains circulating in humans. One of the most powerful and discriminative DNA-based methods for strain typing and population dynamics is the analysis of highly variable co-dominant microsatellite markers. Microsatellites or simple sequence repeats (SSRs) are short, hypervariable, tandemly repeated sequence motifs (1–6 bp), which has evolved and expanded by DNA replication slippage. These may be perfect repeats (consisting of one type of repeat) or might contain single or few base-pair interruptions (1). The genomic regions where microsatellite density (loci/Mbp) is markedly higher than the average density in the genome are called repeat clusters, and those repeating unit which have two or more runs of different repeat motif [e.g. (GTG)8(AT)16] are called compound repeat (2). The rate of mutation of microsatellite rich region is five to six times higher than that of neutral regions of DNA. Tandem repeats (direct or inverted) involved in rearrangements of DNA, alteration of gene copy number (deletion or amplification), formation of extra chromosomal amplicons (circular or linear), and the presence of supernumerary chromosomes have been described in *Leishmania* (3, 4). It has already been reported that *Leishmania* are relatively rich in microsatellites (5). Duhagon *et al.* described non-uniformity of repeat patterns in the intergenic regions, and asymmetrical strand distribution of dinucleotide repeats favoring TT and GT repeats in the coding strands which may control genome structure and gene expression (6). Current analyses of length polymorphism of repeats containing regions shed some light on the population structures and genetic studies of many different species. Recently, multilocus microsatellite typing (MLMT) has been used successfully in *Leishmania* throughout the world to track down different strains and to investigate its population dynamics (7–11). Several studies have discussed the variability of various species of *Leishmania* (12–17). Microsatellite markers designed for *Leishmania* species (13 markers for *L. major*, 16 markers for *L. tropica* and 20 markers for *L. donovani*) have shown high level of polymorphism (18–20). Several microsatellite markers identified in *L. infantum* and others have shown to discriminate between some *Leishmania* populations (21–23). All such studies mainly describe the repeat polymorphism within the same or different species. Despite the medical importance of this parasite, its population genetics is poorly understood. In this respect, the use of molecular markers can provide very useful information for the targeted organisms (24). Moreover, a number of additional applications for the genotype data are possible if the mapped microsatellites with known positions in the genome are used. For example, it is possible to undertake association studies to identify correlations between the frequency of marker alleles and different parasite phenotypes. It is also be possible to search for evidence of recombination within a chromosome (25). But very little is known about the length polymorphism of repeat containing regions. Availability of complete and annotated genome sequences of different *Leishmania* species has provided an excellent opportunity to analyze microsatellites in great detail for their genomic locations, distributions and frequencies. *in silico* mining of microsatellites repeats may provide a useful basis for carrying out further investigation of its structural and functional characteristics. For eukaryotic genome, few such databases for microsatellite searching has been reported in recent years (26–31).

In this article, we describe the development of a microsatellite database (LeishMicrosatDB) using LAMPP (Linux-Apache-MySQL-PHP-Perl) technology, and GenBank of NCBI as a data source to extract the microsatellite data. LeishMicrosatDB is a unique database of microsatellite repeats for diverse *Leishmania* species. The database currently contains 213 chromosomes of six species, and provides information of microsatellite type (simple perfect or compound perfect), repeat unit length (mono- to hexa-nucleotide), repeat number, repeat motif, microsatellite length and chromosomal location in the genome. Furthermore, the information about clustering of different microsatellites and polymorphic repeats (different repeat units of particular loci of different species/strains) can also be retrieved.

## Materials and Methods

### Data source

The chromosome wise genome sequences of six *Leishmania* species and their respective annotation files (.ptt or .gff), available in public domain (ftp.ncbi.nlm.nih.gov/genomes/Protozoa/, http://tritrypdb.org/common/downloads/release-4.1/), were downloaded. The details of each *Leishmania* species are described in Table 1. All possible non-overlapping simple repeats were searched by repeat mining tool called MISA (32). We applied the following criteria (mono—5 repeat unit; di—4 repeat unit; tri to tetra—3 repeat unit and penta to hexa—2 repeat unit) to define each SSR as true repeat. Rationale for choosing the small cutoff value was that, the microsatellites are often disrupted by single base substitution. These simple repeats were mapped on to the genomic annotations from the **.ptt** file using a customized Perl script, **ANNOTATE**. The repeats present within the start and end position of a gene were assigned as coding SSR, and those found in the intergenic regions were considered as non-coding SSR. Left flanking and right flanking sequences (≤250 bp) of each repeat coordinates were extracted by using a perl program called **XTRACT**. For extracting polymorphic repeats, we applied the method described by Pankaj Kumar *et al.* with certain modifications (33), Orthologous parts among the chromosomes were searched using BLASTn using following set of parameters: *E*-value ≤ 0.001; X drop-off value for final gapped alignment = 200; and repeat masking filter = off. Genic and intergenic sequences were screened out by using in-house developed perl script. The repeats were considered as putative Polymorphic Simple Sequence Repeats (PSSR) if a pair of orthologous sequence contains essentially same repeat of different length. To reduce false positives PSSR, left flanking and right flanking sequences of each putative PSSR were compared, and the final PSSR were screened out when identity in corresponding flanking sequences is >60%.

**Table 1.** The details about sequenced *Leishmania* strains, the version of sequenced genomes, annotation status for each genome, number of chromosomes

Serial number	Parasite name	Strain	RefSeq assembly ID	Number of chromosome
1	*L. donovani*	MHOM/NP/2003/BPK282A1	GCF_000227135.1	36
2	*L. infantum*	MCAN/ES/98/LLM-724(JPCM5)	GCF_000002875.2	36
3	*L. braziliensis*	MHOM/BR/75/M2904	GCF_000002845.1	35
4	*L. major*	MHOM/IL/1980/Friedlin	GCF_000002725.1	36
5	*L. tarentolae*	Parrot-TarII	2011-06-22	36
6	*L. mexicana*	MHOM/GT/2001/U1103	2013-01-16	34

## Results and Discussion

### Construction and content of LeishMicrosatDB

**Figure 1.**
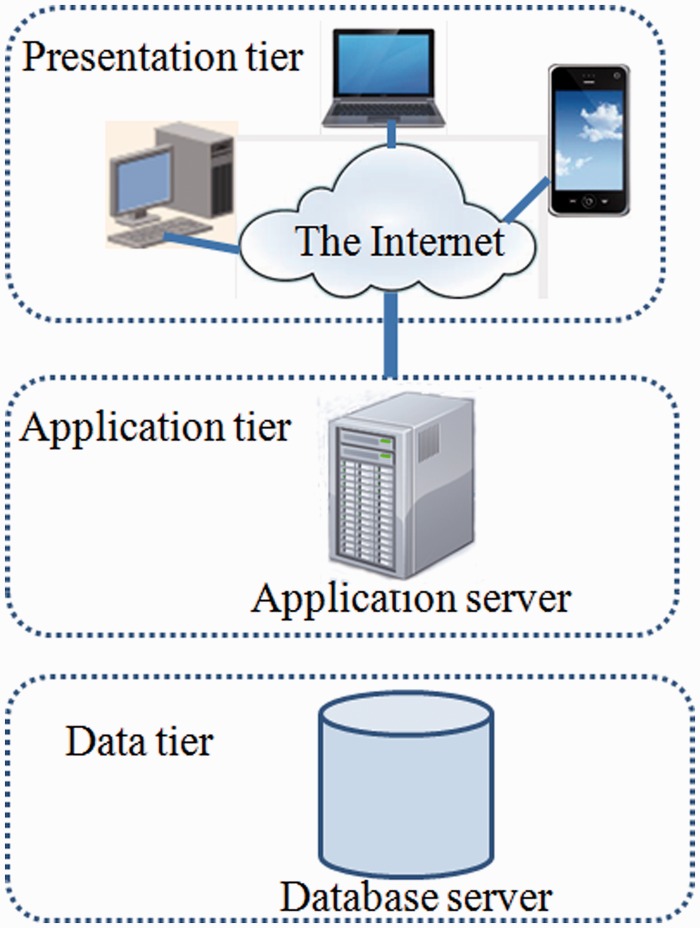
Three tier architecture of LeishMicrosatDB.

**Figure 2.**
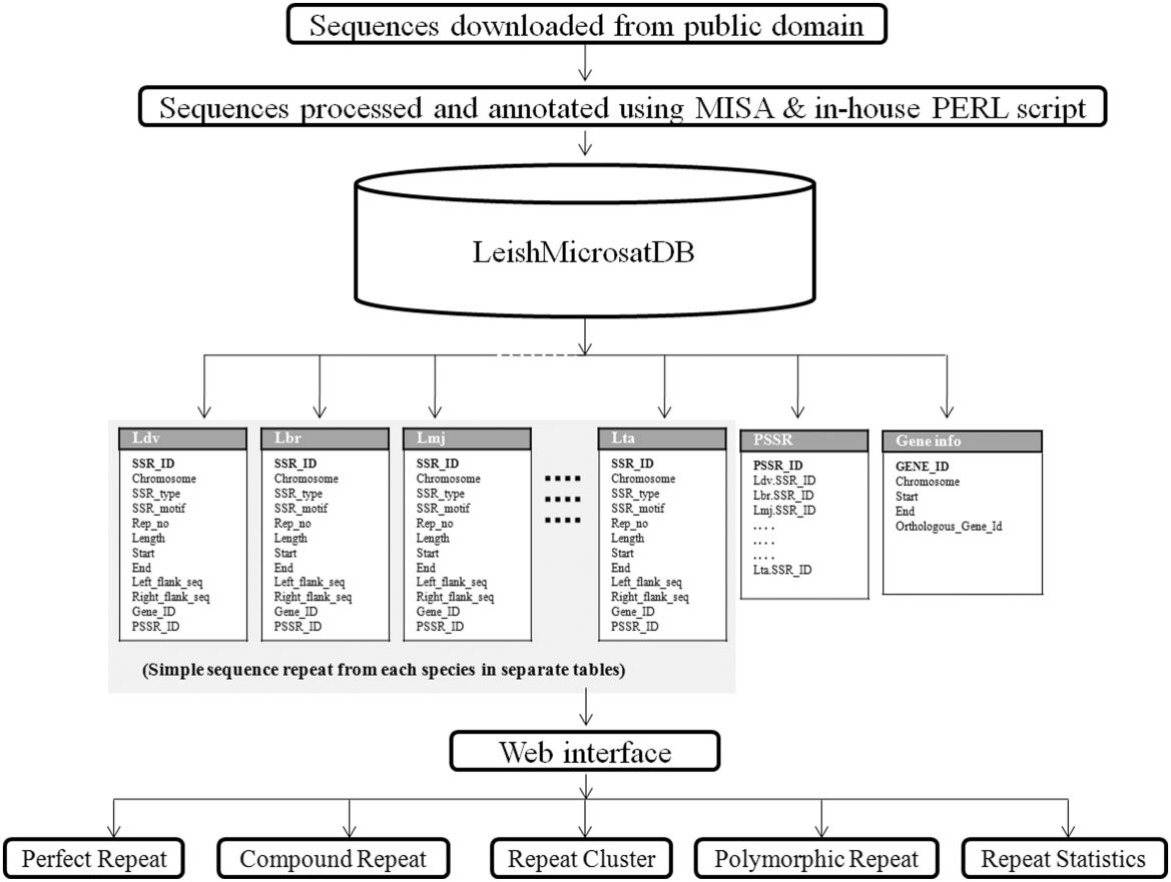
Architecture and data flow representation in LeishMicrosatDB.

In order to manage the data, MySQL, a relational database management system, was used for building the database. A front-end web interface was developed using web technologies like HTML, CSS, JavaScript, DBI (Database Interface), GD (Graphics Design), CGI (Common Gateway Interface) and PERL that communicate with the relational table for data retrieval. The overall architecture of the database is a ‘three-tier architecture’ with a client/presentation tier, middle /application tier and database tier which is outlined in Figure 1. In database tier, tables were designed, and relationships among tables were created using unique, primary and foreign keys. The SSRs identified using MISA from different *Leishmania* species were stored into separate tables. Each species specific table contains field like chromosome, SSR_type, SSR_motif, Rep_no, Length, Start, End, Left_flank_seq, Right_flank_seq, Gene_id and PSSR_ID (Table 2). The PSSR-ID is available for those repeats that are polymorphic. The unique PSSR_ID present in ‘PSSR’ table works as a bridge between individual SSR tables. The Gene table stores genomic coordinate of each gene from each species and its orthologous gene id. This explains the overall schema of the database for efficient data storage and retrieval (Figure 2).

**Table 2.** Structure of the table used in the construction of the LeishMicrosatDB

Field information	Filed name	Data type	Key	Example
Serial number	Sn	Int(20)	PRI	203
Chromosome number	Chromosome	Varchar(2)		11
Repeat type	Type	Varchar(1)		1,2,3,4,5,6
SSR motif	Ssr	Varchar(15)		ACG, GA, AGGCTGA
Repeat number	RepNo	Int(11)		12,10
Total length of the repeated sequence	Length	Int(11)		30,22
Start coordinate of the SSR	Start	Int(10)		10 223, 331 201
End coordinate of the SSR	End	Int(10)		208 871,345 129
Left flanking sequence	Upstream	Varchar(250)		AGGCTAG … AGGTAGC
Right flanking sequence	Downstream	Varchar(250)		AGCtTAG … AGTAGCAA
Gene information if found with in a gene	CodingStatus	Varchar(15)		LTR1234.2, nonCoding,
Polymorphic SSR table Serial Number (if polymorphic)	PSSR_ID	Int(20)		102, 203

### Web visualization of LeishMicrosatDB

LeishMicrosatDB is likely to be accessed by biologist in broad objectives, primarily to develop molecular markers, and also to understand the role of microsatellites in regulating gene expression and genome evolution. The LeishMicrosatDB allows mining of different microsatellites along with their physical location in the chromosomes in six fully sequenced *Leishmania* species. At present, the LeishMicrosatDB has over 1.73 million repeats covering six *Leishmania* genomes. More related genomes will be considered when their whole genome sequences and **.ptt** file be made available in the public domain.

**Figure 3.**
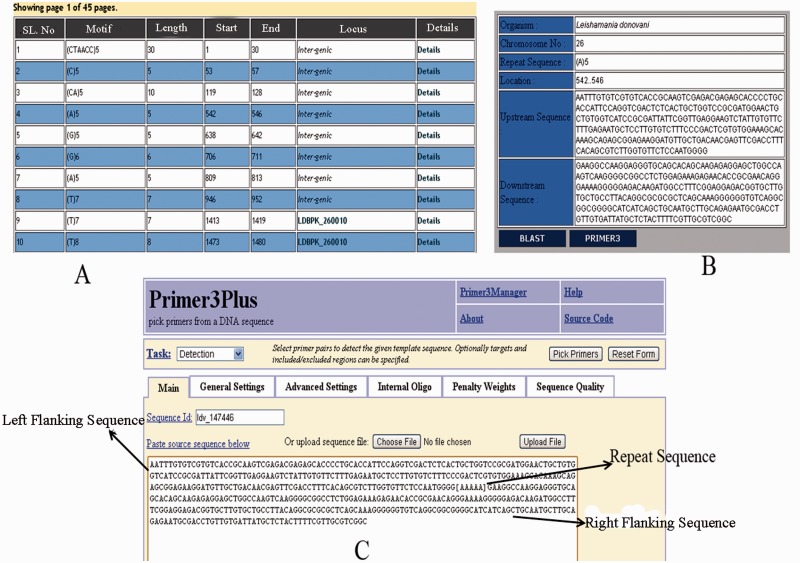
Results displaying repeat information along with left and right flanking sequences and primer3plus primer generation tool.

**Figure 4.**
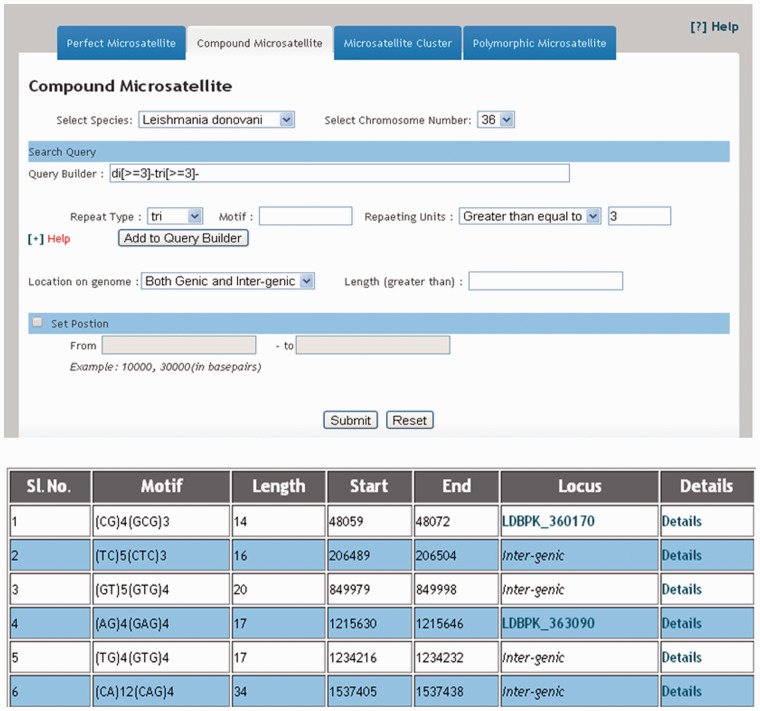
Result displaying compound repeats of any dinucleotide and trinucleotide repeat combination in 36^th^ chromosome of *L. donovani*.

**Figure 5.**
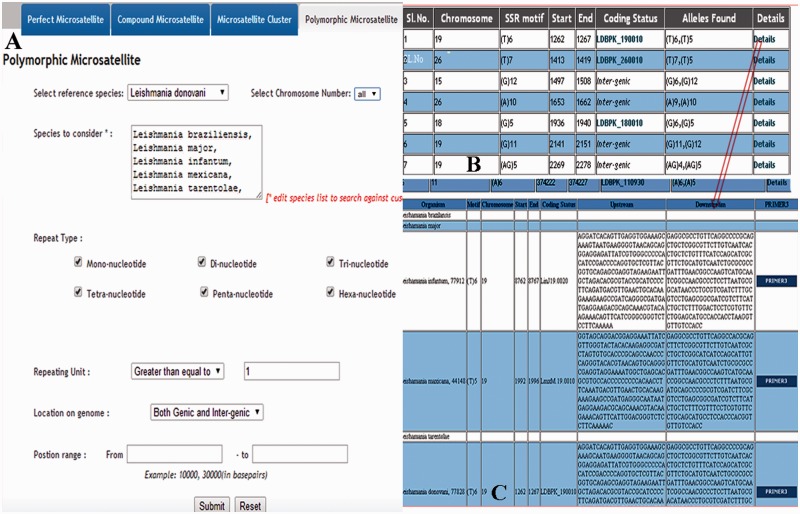
Overview of the retrieving of polymorphic repeats using screen-shots of various pages. (**A**) Main page containing species name which can be selected; (**B**) Overall information of the polymorphic repeats; (**C**) Detail information of the polymorphic repeats.

The web interface of LeishMicrosatDB provides a brief description and links to the page that enables user to select the genome and repeat class of interest. The database can be accessed by perfect repeats, compound repeats, repeat cluster and polymorphic repeats. The perfect repeats can be searched in a chromosome using following need based input parameters likerepeat type (mono- hexa), coding status, repeat unit length and repeat sequence motif. A specific region on the chromosome can be searched by providing input parameters (start and end position). Once species and chromosome options are selected, rest of the fields is set ‘ALL’ by default. The output is primarily a list of microsatellite annotated for all option of the query sheet and the output is generated as a hierarchical pre-sorted list. Each repeat carries its genomic location and corresponding indices. The result page gives complete information of SSR motif, 250 bp left and right flanking sequences that allows user to design locus specific primers. This is facilitated by automatic uploading of repeat and flanking sequences of the selected microsatellite into Primer3 query form (Figure 3). At the bottom of the result page, repeat density map shows the distribution of repeats throughout the chromosome. Apart from the simple sequence repeats or perfect repeats, the database can be accessed for compound microsatellites (two or more microsatellites being found in close proximity) and microsatellite cluster (compound microsatellites interrupted by few nucleotides). Compound repeats can be sought by user’s customized repeat combination. For example, if a user wants to screen compound microsatellites from chromosome 36 of *L. donovani* which has repeat and combination of di- and tri-nucleotide repeat number greater than three unit, search can be made using the parameter specified in Figure 4. Similarly, by specifying the interruption value, the repeat cluster can be accessed. The polymorphic tab contains a drop-down menu comprising the name of all six species. After selecting the target species, rest species were automatically updated in ‘species to consider’ field. A separate option is provided to screen out polymorphic repeats in genic and intergenic regions. The result page contains the number of polymorphic repeats found in the selected species, and gives the detailed information of the particular repeat motif, repeat unit, chromosome number, coding status and genomic location. The output shows information on the corresponding polymorphic repeats (Figure 5). In this page, hyperlinks are also provided to each of the listed polymorphic repeats to design the primers using Primer3. All the detail search methods for perfect repeat, compound repeat, repeat cluster and polymorphic repeats are described in the database tutorial.

**Figure 6.**
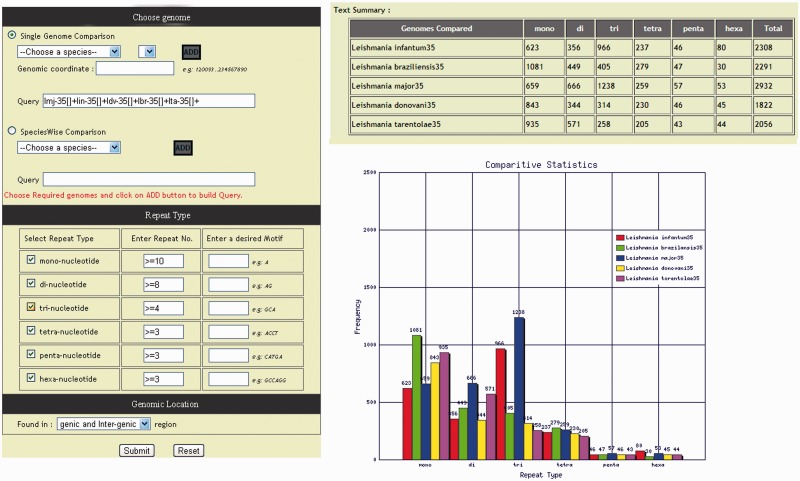
Tabular and graphical representation of microsatellite repeats comparison.

*Leishmania* genomes are varying greatly in microsatellite repeat compositions, diversity and distribution. In order to determine the frequency and composition of different type of repeat motifs available in database, a dedicated section ‘statistics’ has been incorporated in the database which comprises of (i) over all statistics, (ii) a polymorphic SSR statistics and (iii) a comparative statistics, and each statistics can be accessed by a separate ‘tab’. The overall statistics displays chromosome wise over-all repeat statistics of each genome, whereas polymorphic SSR statistics tab displays only the distribution of polymorphic repeats. The comparative statistics tab directs to a repeat summary page giving a detailed illustration of the repeat distribution. The repeat occurrence graph and table are generated dynamically based on the repeat information using GD module (Figure 6). Several microsatellite databases (Table 3) of various organisms have appeared in recent years that provide important data for the comparative analysis of microsatellite distribution in eukaryotic genomes; however, none of these databases provide length variation of SSR across genomes. The LeishMicrosatDB gives useful information such as comparative statistics and length variation across genomes. The identification of polymorphic repeats and its comparative study can exhibit different potential application.

**Table 3.** Comparison of various eukaryotic microsatellite databases, available in public domain

**Database**	**Details on**								**Coverage**
	Simple repeats	Compound repeats	Clustering information	Flanking sequences	Polymorphic information	Genomic repeats	Primer design	Comparative statistics	
MMDBJ (17)	Y	N	N	N	Y	Y	N	N	Mouse
InsatDB (18)	Y	Y	N	Y	N	Y	Y	N	5 Insect genome
MRD (19)	Y	N	N	Y	N	N	N	N	8 eukaryotic genome
SSRD (20)	Y	N	N	Y	N	N	N	N	Human
EuMicrosat*db* (21)	Y	Y	Y	Y	N	Y	Y	N	31 eukaryotic genome
FishMicrosat (22)	Y	Y	Y	N	N	Y	Y	N	36 fish genome
LeishMicrosatDB	Y	Y	Y	Y	Y	Y	Y	Y	6 *L.* genome

## Conclusion

LeishMicrosatDB has been worked out as a complete curated web-oriented relational database of perfect, compound, cluster and polymorphic repeats in six-sequenced *Leishmania* genome. The database can provide parasitologists a platform to understand the diseases by considering the immense utility of the repeats. Various input parameters can be used for comprehensive search of simple, compound, polymorphic and cluster of repeats. This database may also be adopted as a useful tool to study relative occurrence and distribution of microsatellite across the parasitic genome. The repeats in the coding region of the gene may hopefully prove to be more useful for gene tagging and to study its functional role in evolutionary analysis, and all of these information may serve as an important input in designing experiments in new direction, elucidating novel role and function of different kinds of repeats. We anticipate that, the main application of this database will be the development of mapped markers for specific application such as association studies and the search for recombination with in chromosomes.

## Availability

LeishMicrosatDB can be accessed freely at http://biomedinformri.com/leishmicrosat
